# Broadband inhomogeneous lens with conical radiation pattern

**DOI:** 10.1038/s41598-023-40024-9

**Published:** 2023-08-09

**Authors:** Mohammad Mahdi Taskhiri, Saeed Fakhte

**Affiliations:** Department of Electrical and Computer Engineering, Qom University of Technology, Khodakaram Blvd, Old Qom-Tehran, Road, Qom, 1519-37195 Iran

**Keywords:** Electrical and electronic engineering, Optical sensors

## Abstract

This manuscript presents a lens antenna with simultaneous broadside and conical beams. The lens is designed for the Ku band using the ray inserting method. The proposed conical radiation pattern is broadband due to good matching with the source and surroundings. The simulation is conducted using the CST microwave studio solver. Instead of complex antenna shapes used in other works, a simple circular patch ring and RF connector are used as the lens feed to generate broadside and omnidirectional conical patterns, respectively. To validate the performance of the designed lens and its two-port feed antenna, the lens structure is realized and fabricated using the 3D printing method. Polyethylene terephthalate glycol (PETG) plastic material is utilized for constructing the lens in this work. The electromagnetic characteristics of PETG in the Ku band are accurately measured. The results of simulations and experiments demonstrate the good performance of the designed lens over a wide frequency bandwidth. The advantage of this designed structure over other works is its high gain and broad bandwidth.

## Introduction

High-speed wireless data communication is required for many applications that implicate ultra-wideband and high-gain antennas. In recent years, conical radiation pattern antennas have been used in various applications such as high-speed data transfer, automotive, side lobe canceler in radars, medical sensing, satellite reception, and mobile communication^1–7^. The conical beam radiation pattern is valuable for applications independent of a horizontally omnidirectional radiation pattern to provide the best performance-to-cost ratio, such as satellite tracker systems^4,8^. Recently some research concentrated on the Ku-band frequency range for high-frequency communication systems^9^. Systems with two broadside and conical beams have been developed in simultaneous^10^ or switchable feed^11^ antennas. To increase the reliability of a communication system, pattern diversity antennas can be considered at the transceiver^12,13^.

Recently, different methods have been proposed to design a wideband conical pattern antenna, such as some shapes of the planar monopole^14–16^, substrate-truncated microstrip circular patch^17^, open-ended coaxial waveguide^18^, mode conversion in a circular waveguide^19^, and radially arrayed slot antenna^10,20^. Different resonant modes of circular patch antennas can be excited to radiate broadside and conical patterns^21,22^. Adopting two separate ports with different radiating modes for a single patch antenna is another method to have pattern diversity^23^.

There are several methods to have an antenna with broadband and high directivity radiation beam. Planar transmit-arrays and reflectarrays designed based on frequency selective surfaces (FSS)^24,25^ or homogeneous^26^ and inhomogeneous^27^ dielectric are some methods to achieve high-gain radiation patterns. The complexity of construction, limited bandwidth, and the realization of multiple separate layers are some of the main drawbacks of metamaterial-based methods. In recent years, several methods have been introduced to improve the performance of metamaterial structures. The optimal performance and simplicity of manufacturing processes are some of the advantages of metamaterial structures^28,29^. Introduces separate phase profiles in two circular polarization-preserving channels, allowing for separate holographic images in two polarization-preserved fields with different propagating distances. It is designed with five metallic layers separated by four dielectric layers and is passive, lossless, and reciprocal. Dielectric lenses are another suitable option to collimate the beam of the feed source in the front direction or shape the desired radiation pattern^30–33^.

The inhomogeneous lenses are divided into isotropic and anisotropic. Usually, anisotropic lenses are designed based on the transformation optics (TO) method^34–37^. This is a new technique to realize novel structures and applications while maintaining the original electromagnetic performances, which were unattainable previously. These types of lenses have a complexity of construction and bandwidth limitations. Various techniques have been proposed to eliminate these problems^38,39^. Reference^39^ discusses the trade-off between the required material parameters and the desired performance of the device. The lens in this study is constructed using non-resonant cells, with air holes drilled in a dielectric host medium.

The ray insertion method (RIM) is an approach to bending wave manipulation^40^. It is an accurate analytical method based on geometrical optics by providing a parametric ray path relation for designing various inhomogeneous structures such as lenses^41,42^, bends^40^, beam splitters^43^, and concentrators^44^. Electromagnetic structures designed using this method have two main features: realisability and broad bandwidth.

The effective-medium theory can be used by embedding air holes in cubic or fan-shaped dielectric unit cells to realize inhomogeneous permittivity distribution. 3-D Printed techniques (additive manufacturing)^34,45,46^, printed metasurfaces^47,48^, laser-cutting^49^, PCB milling, and dielectric substrate drilling^50,51^ are methods to fabricate this medium.

In this article, an inhomogeneous lens is proposed based on the ray-inserting method. A simple circular patch ring and RF connector are used as a feed of the lens to generate broadside and omnidirectional conical patterns, respectively. The presented inhomogeneous lens not only increases the gain of the RF connector monopole antenna and creates a conical-shaped radiation pattern but also can develop a suitable matching in the proper frequency bandwidth. The Fullwave CST simulation is used to validate the design lens antenna. The isolations between the two ports of the antenna are higher than 20 dB, which is considerable for pattern diversity applications. A prototype is fabricated and tested. Good agreement is observed between the simulated and measured radiation patterns.

Many methods have been introduced to design lens antennas to radiate directive broadside radiation patterns, and the proposed design method based on the ray inserting method (RIM) is used to design a conical radiation pattern inhomogeneous lens. The advantage of the presented designed structure over other works is its high realized gain and broad bandwidth. Also, the proposed design method is simple and uses a circular patch ring and RF connector as a feed of the lens to generate broadside and omnidirectional conical patterns, respectively, instead of complex antenna shapes in other works.

The manuscript is structured as follows. The lens design procedure based on the ray-inserting method is discussed in “Conical pattern lens design using RIM equations” section. Also, the feed antenna is designed in this section. In “Realization and fabrication of the lens profile” section, the designed lens is realized and fabricated. The performances of the designed lens are discussed in “Simulation and measurement results” section. Finally, a conclusion is offered in “Conclusion” section.

## Conical pattern lens design using RIM equations

Many methods have been introduced to design lens antennas to radiate directive broadside radiation patterns. This section aims to concentrate on the design of a conical radiation pattern inhomogeneous lens. Lissajous curves are a family of curves similar to an ellipse, described by parametric equations^52^. The RIM equation is the developed model of the Lissajous equation, in which the constraints of the Eikonal equation^53^ are taken into account. The Eikonal equation describes a relationship between the refractive index of a medium and the ray’s path trajectory based on geometrical optics. The eikonal equation in the inhomogeneous, isotropic, and lossless medium is the form

1 $$\begin{aligned} {n^2}(\rho ,z) = (d\rho /dt)^{2} + (dz/dt)^{2} \end{aligned}$$

where $n = \sqrt{{\varepsilon _r}}$ is the refractive index of the medium. Based on Eikonal equations, longitudinal and transverse changes in a gradient refractive index structure cause wave flexural. The effective optical length of each ray is independent of frequency, $\omega$, and is defined as ${l_e} = \frac{{c\Delta \phi }}{\omega } = \int _0^{{t_f}} {n\,.dl} = \int _0^{{t_f}} {{n^2}(t)\,dt}$, where the variable *t* varies from 0 to $t_f$ for input and output surfaces, respectively. The parameter $t_f$ is the final value of the parameter *t* and may be different from ray to ray.

**Figure 1:**
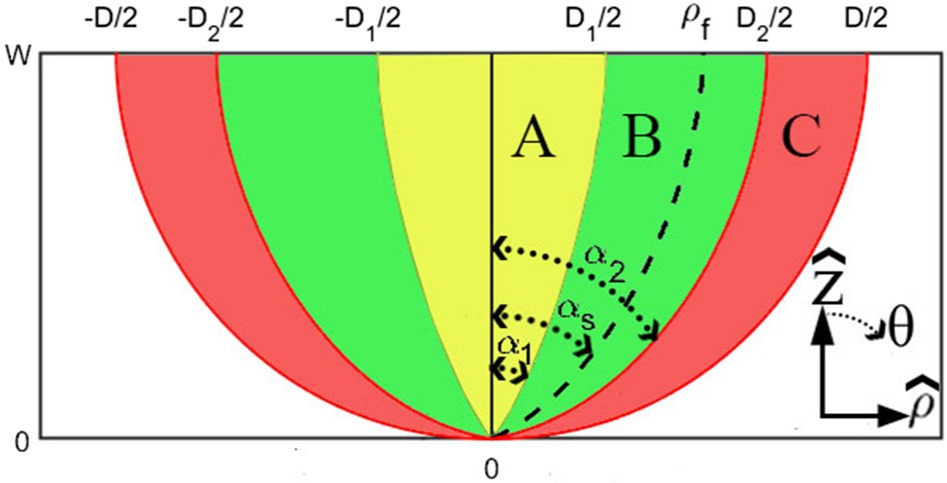
A typical configuration of a conical radiation pattern inhomogeneous lens antenna.

Equation 2 is the generic form of the RIM equation, which has unknown constants determined using known parameters at the beginning and end points of the rays. The nonlinear RIM equation describes the ray path and its particular refractive index.

2 $$\begin{aligned} \rho (t)= & {} {a_\rho }\sin \left( {{k_\rho }t} \right) + {b_\rho }cos\left( {{k_\rho }t} \right) + {c_\rho }\nonumber \\ z(t)= & {} {a_z}\sin \left( {{k_z}t} \right) + {b_z}cos\left( {{k_z}t} \right) + {c_z} \end{aligned}$$

where ${a_\rho },\mathrm{{ }}{\mathrm{{a}}_z},\mathrm{{ }}{\mathrm{{b}}_\rho },\mathrm{{ }}{\mathrm{{b}}_z},\mathrm{{ }}{\mathrm{{c}}_\rho },\mathrm{{ }}{\mathrm{{c}}_z},\mathrm{{ }}{\mathrm{{k}}_\mathrm{{\rho }}},\,{k_z}$ and ${t_f}$ are unknown constants. The variable t varies from 0 to $t_f$ for each ray’s input and output points, respectively. Figure 1 shows a typical configuration of a conical radiation pattern inhomogeneous lens antenna. A sample inserted ray emits from point (0, 0) with an $\alpha _s$ angle and with a refractive index of One, and after traveling inside the lens, reaches the point ($\rho _f$, *W*) at an angle of 0 degrees with a refractive index of One. ${\alpha _s}$ varies from $-90^{\circ}$ to $90^{\circ}$. The rays are ended in the normal direction of an aperture with a diameter of *D*. To create a high-gain conical pattern, the optical length of all rays should be equalized to the constant value ($l_{e}$). The maximum permittivity is directly dependent on the value of $l_{e}$. These parameters lead to the determination of unknown values in the parametric RIM equation. After some mathematical manipulations, the parameters $k_\rho$ and $k_z$ could be found from the following nonlinear relations:

3 $$\begin{aligned} \textrm{tan}(\textrm{k}_{\rho }\textrm{t}_{\textrm{f}}/2)= & {} \frac{\rho _{\textrm{f}}}{\textrm{sin}(\alpha _{\textrm{s}})}{\textrm{k}}_{\rho } \nonumber \\ \textrm{tan}(\textrm{k}_{z}{\textrm{t}}_{\textrm{f}}/2)= & {} \frac{\textrm{W}}{1 + \textrm{cos}(\alpha _{\textrm{s}})}\textrm{k}_{z} \end{aligned}$$

The trajectory of the rays versus an independent variable t can be simplified as follows:

4 $$\begin{aligned} \rho (t)= & {} (\mathrm{{sin(}}{\alpha _\mathrm{{s}}}\mathrm{{)/}}{\mathrm{{k}}_\rho })\sin \left( {{k_\rho }t} \right) + \frac{{{\rho _\mathrm{{f}}}\mathrm{{ - }}\frac{{\mathrm{{sin(}}{\alpha _\mathrm{{s}}}\mathrm{{)sin(}}{\mathrm{{k}}_\rho }{\mathrm{{t}}_\mathrm{{f}}}\mathrm{{)}}}}{{{\mathrm{{k}}_\rho }}}}}{{\mathrm{{cos(}}{\mathrm{{k}}_\rho }{\mathrm{{t}}_\mathrm{{f}}}\mathrm{{) - 1}}}}(cos\left( {{k_\rho }t} \right) - 1) \nonumber \\ z(t)= & {} (\mathrm{{cos(}}{\alpha _\mathrm{{s}}}\mathrm{{)/}}{\mathrm{{k}}_z})\sin \left( {{k_z}t} \right) + \frac{{\mathrm{{W - }}\frac{{\mathrm{{cos(}}{\alpha _\mathrm{{s}}}\mathrm{{)sin(}}{\mathrm{{k}}_\mathrm{{z}}}{\mathrm{{t}}_\mathrm{{f}}}\mathrm{{)}}}}{{{\mathrm{{k}}_z}}}}}{{\mathrm{{cos(}}{\mathrm{{k}}_\mathrm{{z}}}{\mathrm{{t}}_\mathrm{{f}}}\mathrm{{) - 1}}}}(cos\left( {{k_z}t} \right) - 1) \end{aligned}$$

**Figure 2:**
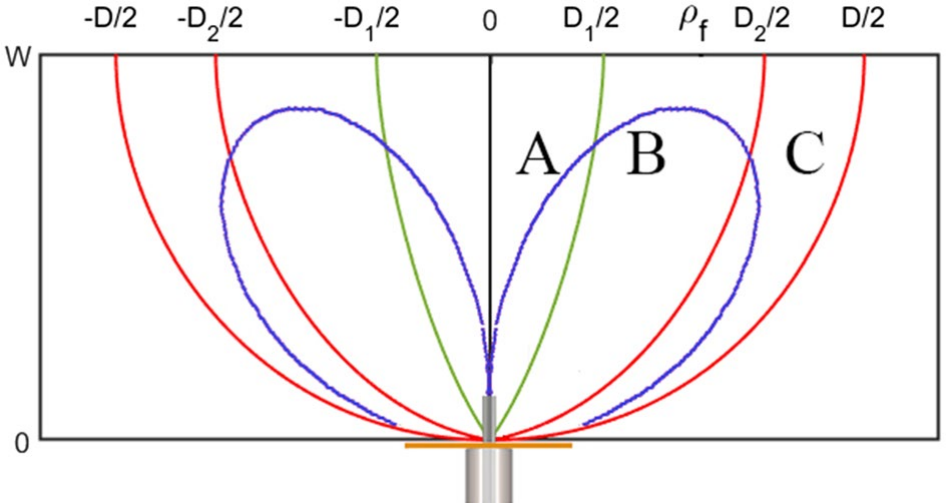
An orientation of a lens and monopole feed and a typical radiation pattern of the monopole antenna.

A simple surface mount RF coaxial connector, i.e., SMA (subminiature version A), monopole antenna with omnidirectional radiation is considered the lens’s feed to radiate a conical-shaped pattern. Figure 2 shows an orientation of a lens, monopole feed, and a typical radiation pattern of the monopole antenna. The radiation of monopole feed at $\theta =0^{\circ}$ is near zero. The density of radiated electric field increases significantly for angles greater than zero degrees. As seen in Fig. 1, the lens is divided into three sections A, B, and C. To have a more directive conical-shaped radiation pattern, the size of section B should be optimized. In other words, the endpoint of rays ($\rho _f$) with intermediate radiated angles should be given more attention. Therefore, the lens should be able to lead the radiation field to greater angles than $\theta =0^{\circ}$. The omnidirectional radiation pattern of the monopole feed antenna facilitates this phenomenon.

**Figure 3:**
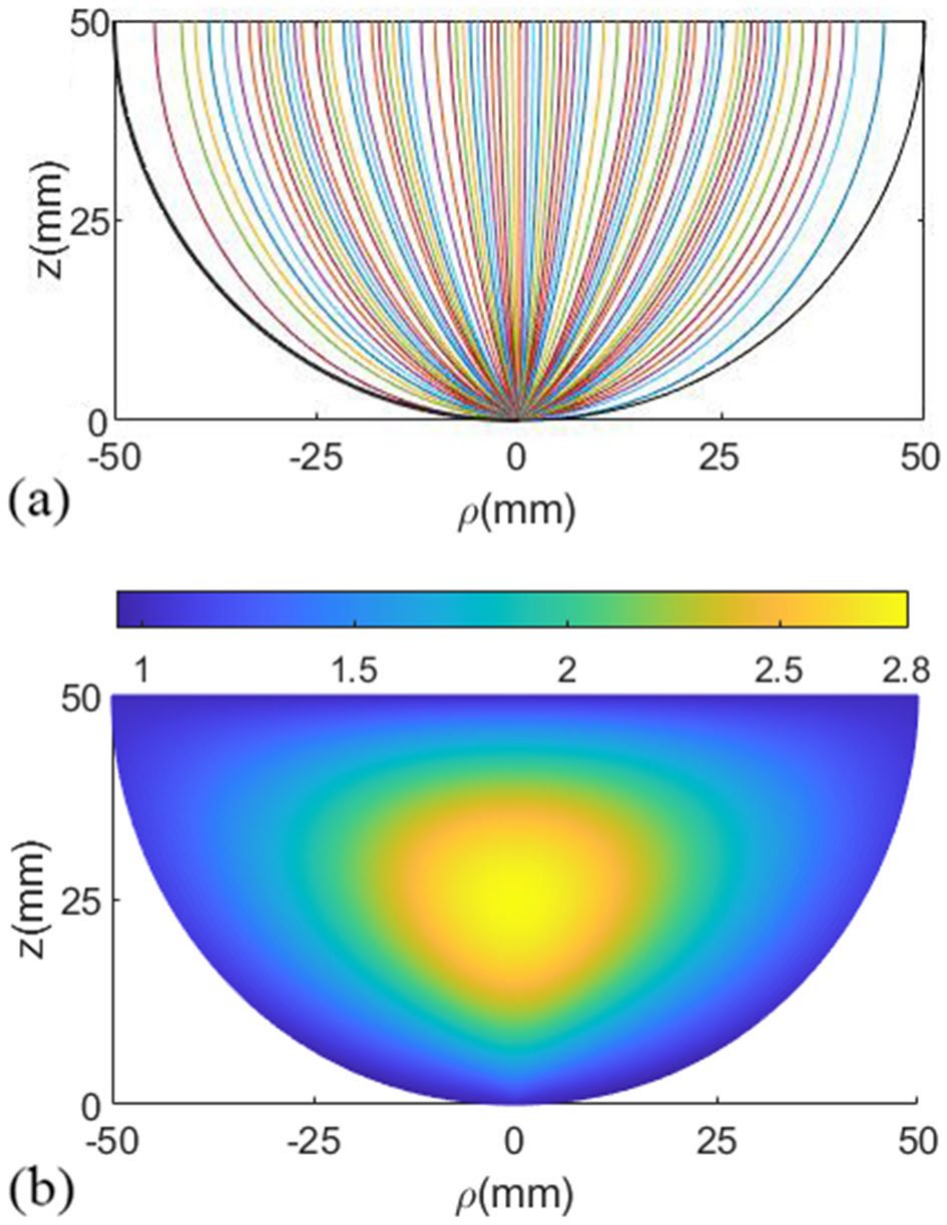
(**a**) The rays’ path and (**b**) relative permittivity distributions of the designed lens.

**Figure 4:**
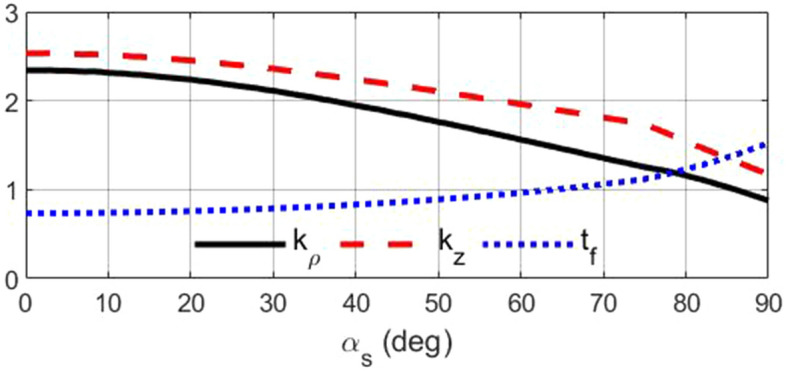
The values of unknown parameters for the designed optimized lens.

At first, the lens dimensions are considered for operation in the Ku-band, i.e., $F=50$ mm and $D=100$ mm. Uniform endpoints distribution is considered in each section. To have maximum conical pattern directivity, $D_1$, $\alpha _1$, $D_2$, and $\alpha _2$ should be optimized. We utilized the CST Studio Suite to MATLAB Interface to optimize the geometry of the lens structure. The primary objective was to manage the CST project, retrieve and analyze the simulation results, export the geometry, and obtain various information from the project. The lens geometry’s programmable capability has facilitated the contribution to the Matlab File Exchange. The maximum gain of the conical pattern was regarded as the goal function. After optimization, the value of $D_1=10$ mm, $\alpha _1=10^{\circ}$, $D_2=65$ mm, and $\alpha _2=65^{\circ}$ are obtained. The electrical length is optimized to have maximum permittivity of 2.8, i.e., $l_{e}=2.6$. The rays’ path and relative permittivity distributions are shown in Fig. 3. The refractive index is ended to one on the output plane, so the lens is matched to the surrounding space. The plots of unknown parameters are shown in Fig. 4 for the optimized lens.

Also, a simple circular patch ring is designed to feed the lens at the center frequency. This feeding cause a directive broadside radiation pattern. The lens should be optimized for a better conical radiation pattern. A directive broadside radiation pattern can be obtained from this designed lens.

**Figure 5:**
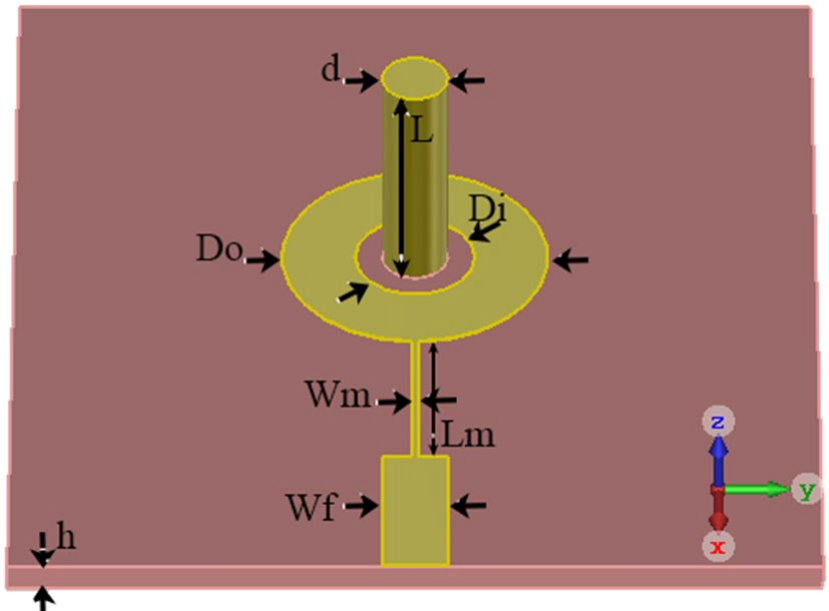
The geometry of the proposed feeding antenna.

**Figure 6:**
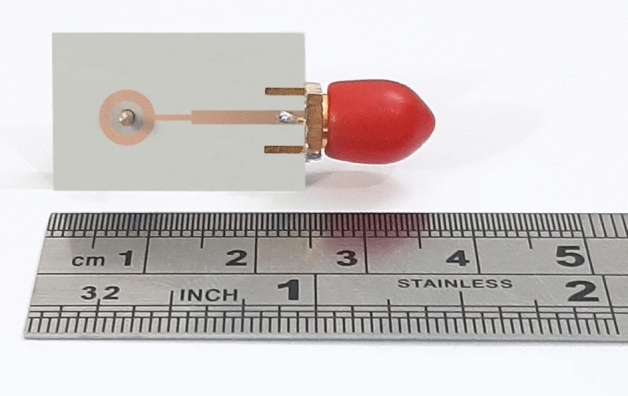
The fabricated antenna.

The geometry of the proposed feeding antenna is shown in Fig. 5. The optimized physical dimensions of the feeding structure are included in Table 1. The antenna is fabricated on a substrate of Rogers R04003C with a thickness of 20 mil and $\epsilon _r$ = 3.38 (see Fig. 6). Both ports are matched to the $50 \,\Omega$ SMA connectors.

**Table 1:** The optimized physical dimensions of the feeding antenna.

Parameter	*h*	*L*	$W_f$	$W_m$	$L_m$	$D_i$	$D_o$	*d*
Value (mm)	0.508	3.2	1.17	0.108	3.26	2	4.47	1.2

## Realization and fabrication of the lens profile

To ensure the performance of the designed lens and its two port feed-antenna, the structure of the lens is realized and fabricated using the 3D printing method. Fused deposit manufacturing (FDM) is one of the most widely used 3D printing methods. Extruding a melted material through a nozzle is done in the FDM method to build an arbitrary structure. The most commonly used materials for 3D printing are acrylonitrile butadiene styrene (ABS), polylactic acid (PLA), and polyethylene terephthalate glycol (PETG). PETG has sufficient ultimate strength and stiffness. The advantages of PETG are durability and good layer adhesion^54^. The nozzle temperature during the printing of PETG filament is 240 °C which is higher than recommended value for PLA. High impact resistance, heat resistance, relatively low cost, fully recyclable, and water resistance make PETG the best choice for 3D printing filament options^55^. So, PETG plastic material is utilized to construct the lens in this work.

**Figure 7:**
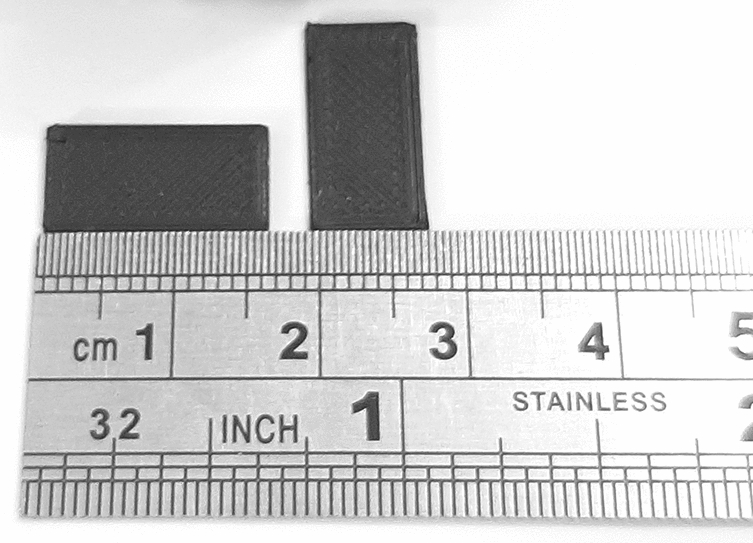
The PETG sample.

**Figure 8:**
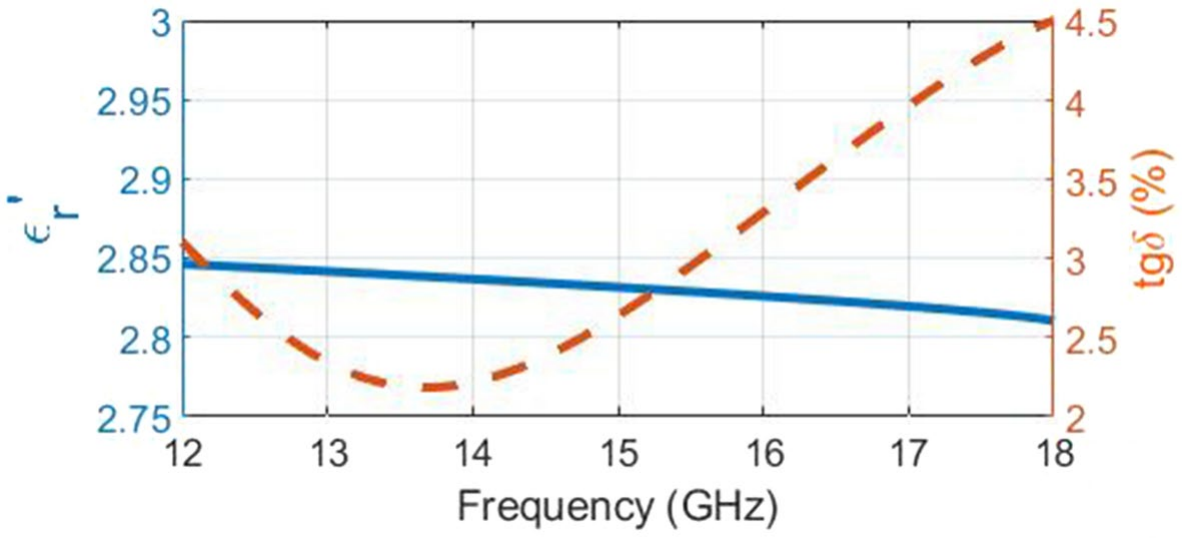
Measured results of relative permittivity (dashed line) and $tg\delta (\%)$ (dotted line) of PETG.

The electromagnetic characteristic of PETG must be accurately determined for the optimal performance of the lens in the microwave frequency band. Different colors of the material filament may be determinative in the value of its dielectric coefficient^56^. Both permittivity and permeability can be directly calculated by Nicholson–Ross–Weir (NRW) method^57^ from the s-parameters. Rectangular waveguide with a long wall length of 15.8 mm and width of 7.9 mm, samples of a 15.8 mm length, width of 7.9 mm and thickness of 1 mm, and the network analyzer Rohde and Schwarz (ZVA series) are used to measure the complex permittivity value of PETG. Figure 7 shows the PETG sample. To ensure the maximum possible homogeneity and density of the sample’s internal structure, it was printed at a resolution of 50 μm and a fill density of $100\%$. Figure 8 shows the measurement result of sample PETG material in the Ku-band waveguide test fixture. The measured $\epsilon _r'$ and loss tangent are 2.83 and 0.026 at 15 GHz, respectively. The measurement results of the PETG dielectric coefficient reported in different articles are presented in Table 2.

**Table 2:** The measurement results of the PETG dielectric coefficient reported in different articles.

Refs.	Real part of permittivity	$tg\delta (\%)$	Ref. frequency range
^58^	3.3	1.24	1–100 [MHz]
^59^	2.87	2.43	1–10 [GHz]
This work	2.83	2.6	12–18 [GHz]

The effective medium theory is considered to realize the inhomogeneous permittivity of the proposed lens. The permittivity smoothly changes from One on the outer side sections to a maximum value of 2.8 at the center section of the lens (see Fig. 3).

**Figure 9:**
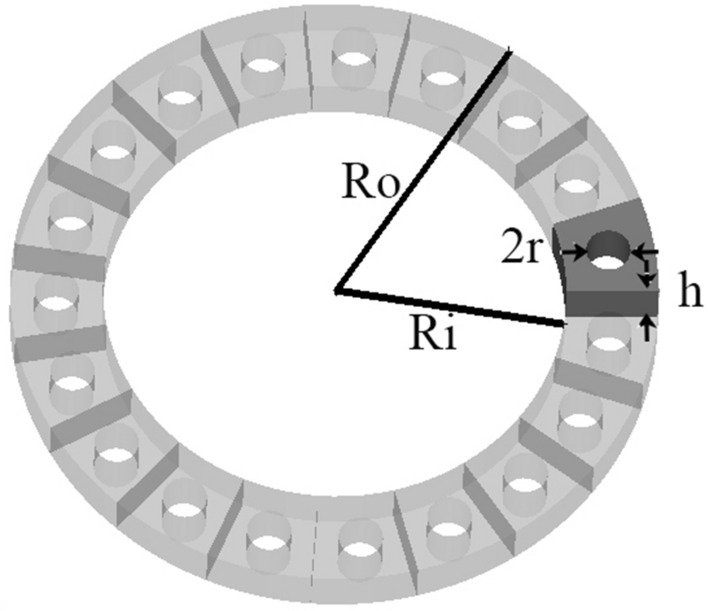
A model of fan-shaped unit cells.

Figure 9 shows a model of fan-shaped unit cells that completes a ring structure by being placed next to each other. The background PETG material is perforated with circular holes. The number of fan-shaped cells should be an integer in a ring with a radius of $R_c$ and is calculated as follows.

5 $$\begin{aligned} \mathrm{{N = round}}(\pi ({\mathrm{{R}}_\mathrm{{o}}}\mathrm{{ + }}{\mathrm{{R}}_\mathrm{{i}}}\mathrm{{)/}}({\mathrm{{R}}_\mathrm{{o}}}\mathrm{{ - }}{\mathrm{{R}}_\mathrm{{i}}})) \end{aligned}$$

where $R_o$ and $R_i$ are equal to the outer radius and the inner radius of the unit cell, respectively. The effective permittivity of a perforated unit cell is

6 $$\begin{aligned} {\varepsilon _{re}} = {(\sqrt{{\varepsilon _{r}}} (1 - \eta ) + \eta )^2} \end{aligned}$$

where $\eta$ is the filling factor of the unit-cell area. The filling factor is defined as the ratio of the hole size to the unit-cell area.

7 $$\begin{aligned} \eta = \frac{{{S_{circular\,hole}}}}{{{S_{unit\,cell}}}} \end{aligned}$$

where $S_{circular\,hole}$ is the area of the air hole, and $S_{unit\,cell}$ is the area of the lattice unit cell.

8 $$\begin{aligned} {S_{unit{\hspace{1.0pt}} cell}}= & {} \pi ({R_o}^2 - {R_i}^2)/N\nonumber \\ {S_{circular\,hole}}= & {} \pi {r^2} \end{aligned}$$

**Figure 10:**
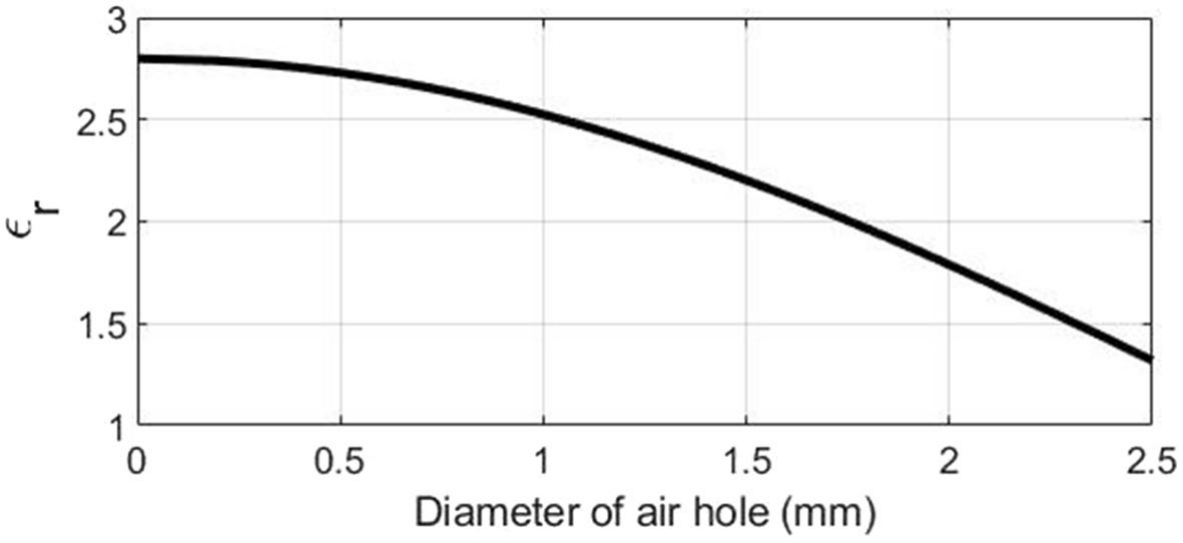
The variation of effective permittivity of a fan-shaped unit-cell with different hole diameters.

**Figure 11:**
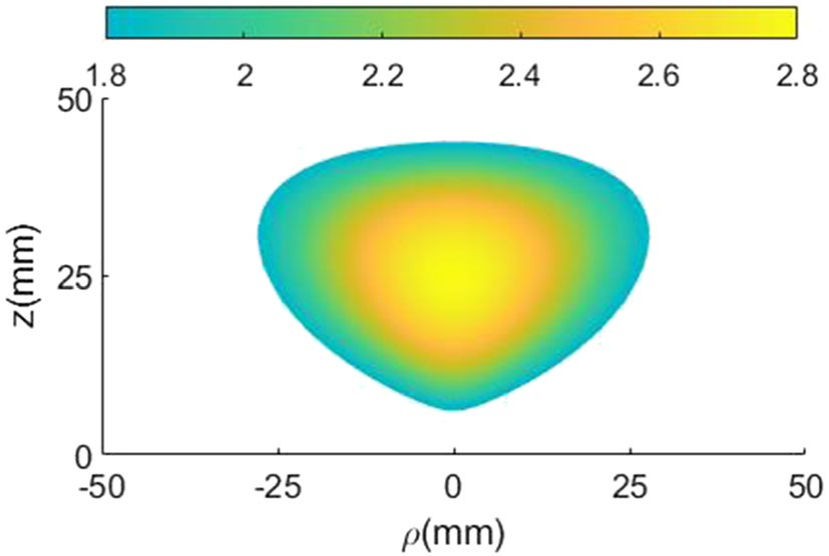
The two-dimensional cut of the permittivity distribution of the designed lens in the range of 1.8–2.8.

By substituting the area of the circular hole and the area obtained from Eq. (8) in Eq. (6), the hole’s radius (*r*) can be obtained. The difference between the outer and inner radius of rings ($R_o-R_i$) is considered 2.5 mm ($=0.125\lambda _c$), and its height is limited to 1.5 mm ($=0.075\lambda _c$), where $\lambda _c$ is the wavelength of the center frequency (15 GHz). The variation of effective permittivity of a unit-cell element with different hole sizes for PETG plastic materials is shown in Fig. 10. To fabricate the designed lens using FDM technology, the constraint of the minimum thickness of 0.5 mm for the unit cell’s walls should be fulfilled. Therefore, the lowest achievable effective relative permittivity using circular air holes is 1.8. Figure 11 shows the two-dimensional cut of the permittivity distribution of the designed lens in the range of 1.8–2.8. According to the path of rays in Fig. 3, section C in Fig. 1, and the omnidirectional pattern of the SMA feed antenna in Fig. 2, it can be seen that the part of the lens whose permittivity is less than 1.8 (section C) has a minor effect on the final result and can be neglected.

**Figure 12:**
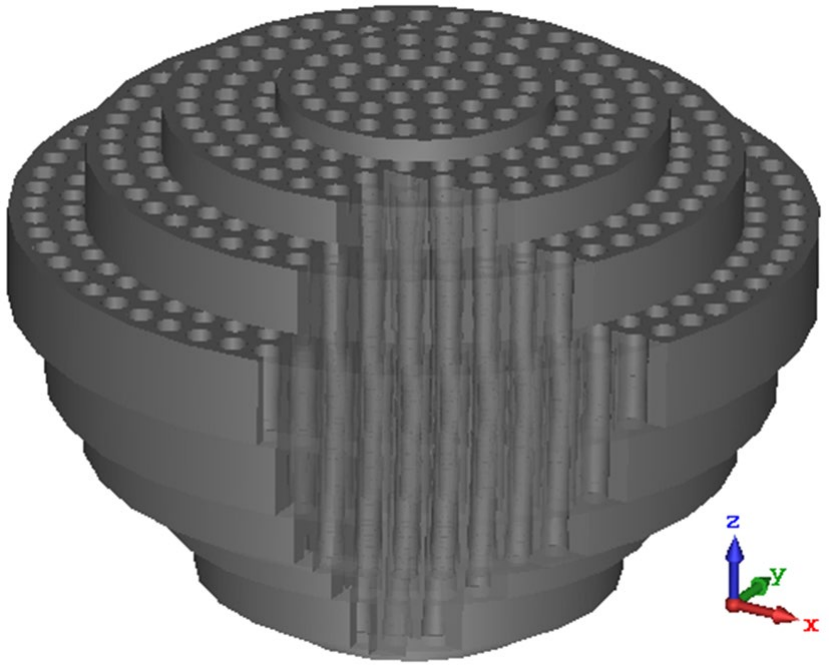
The schematic of the realized graded lens.

**Figure 13:**
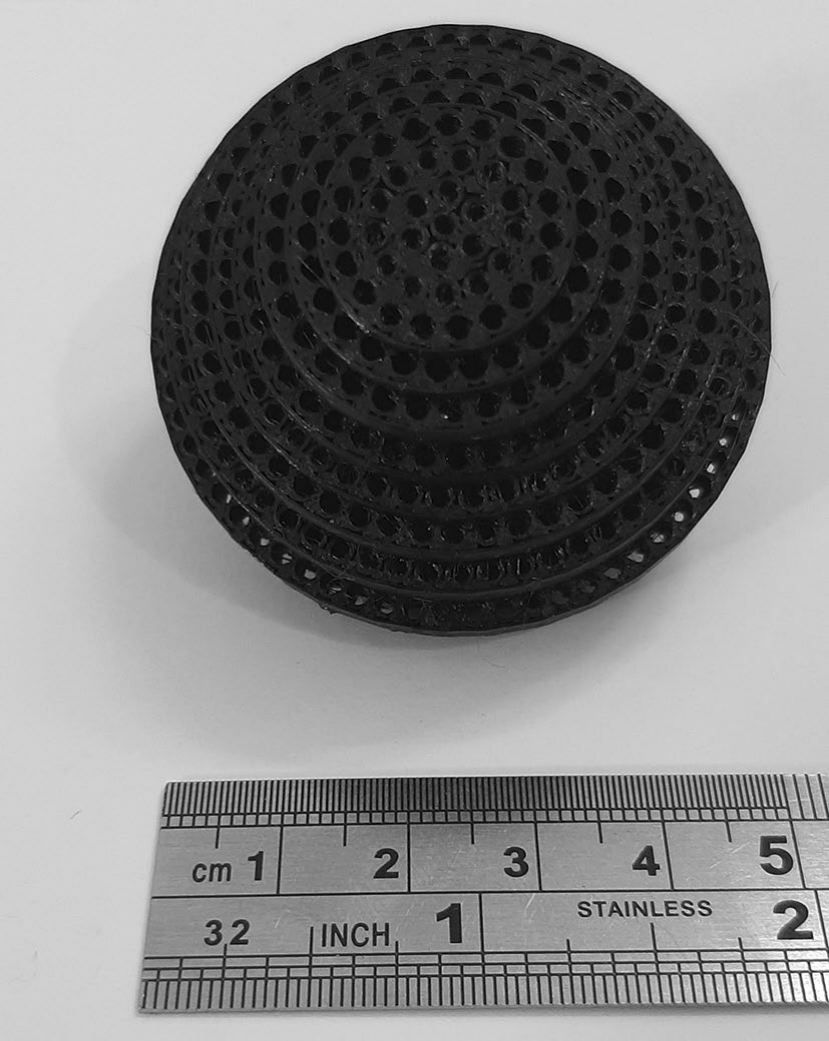
The fabricated lens model, contains 5100 cells.

The lens with a simple two-ports feed-antenna has been designed, fabricated, and tested based on the derived equations. The schematic of the realized graded lens is shown in Fig. 12. Figure 13 shows the fabricated lens model, which contains 5100 cells. The diameter and height of the 3D-printed lens are $D=50$ mm and $W=37.5$ mm, respectively. It is seen that the hole’s diameter increases from the center toward the sides of the lens. The weight of the printed lens is 41.5 g.

## Simulation and measurement results

**Figure 14:**
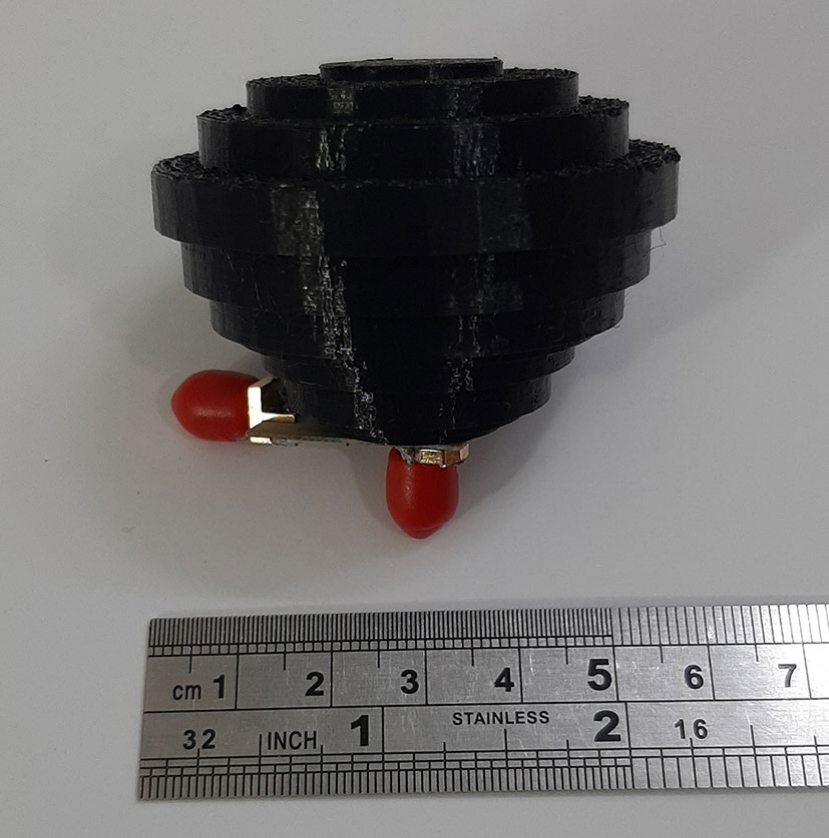
The set of fabricated antenna and lens.

**Figure 15:**
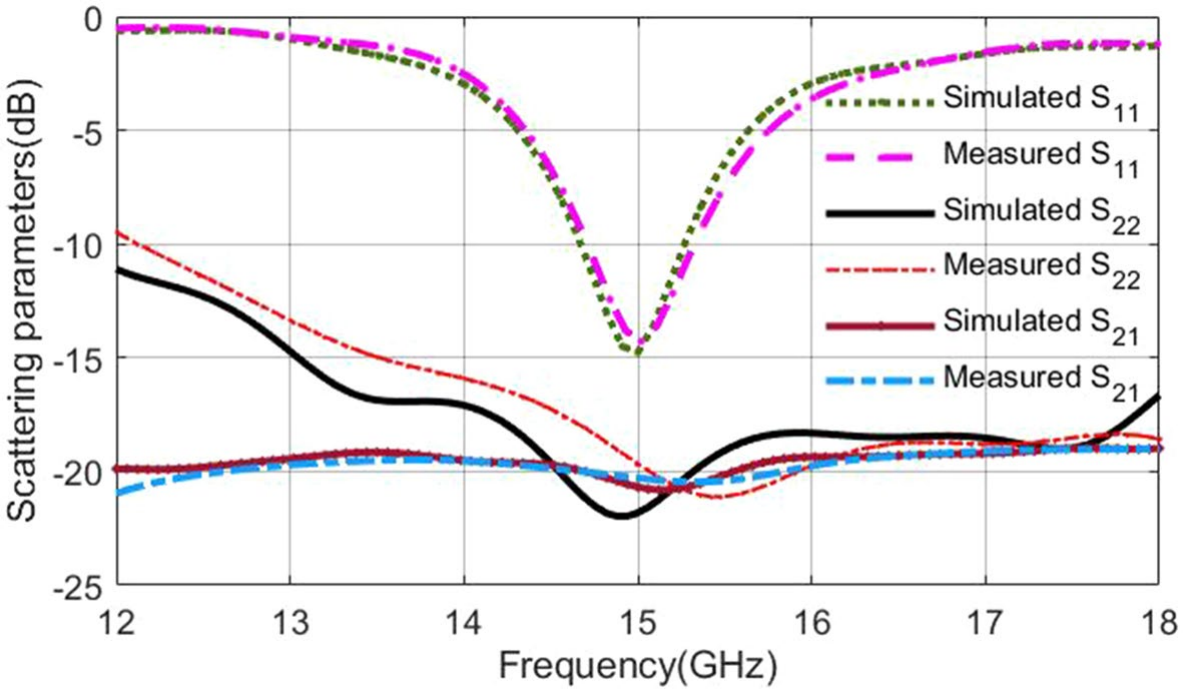
The reflection coefficients and isolation of the ring patch and monopole antenna ports. The port of the ring patch is considered port one, and the port of SMA is selected as the second port.

The set of fabricated antenna and lens is shown in Fig. 14. In this section, the port of the ring patch, which radiates a broadside pattern, is considered port one, and the port of SMA, which radiates a conical pattern, is selected as the second port. The reflection coefficients and isolation of the ring patch and monopole antenna ports are shown in Fig. 15, respectively. A single SMA acts like a monopole antenna with limited frequency bandwidth and omnidirectional radiation pattern. The SMA pin height (parameter *L* in Fig. 5) was optimized to cover the maximum possible bandwidth. As can be seen, the reflection coefficient of $S_{22}<-10$ dB covers the frequency range of the Ku-band due to the proper matching between the lens and the coaxial feeding probe.

The ring patch has a limited bandwidth due to its resonant behavior. Various conventional antennas designed in different papers (mentioned in the introduction) could be chosen to feed the proposed inhomogeneous lens. But the primary goal of this manuscript is to innovate a broadband conical-shaped radiation pattern lens, and having a broadside radiation pattern is a side benefit of this lens. The port-to-port isolation is essential in pattern diversity application. Figure 15 depicts that the isolation between two ports is higher than 20 dB, which is considerable for pattern diversity antennas.

**Figure 16:**
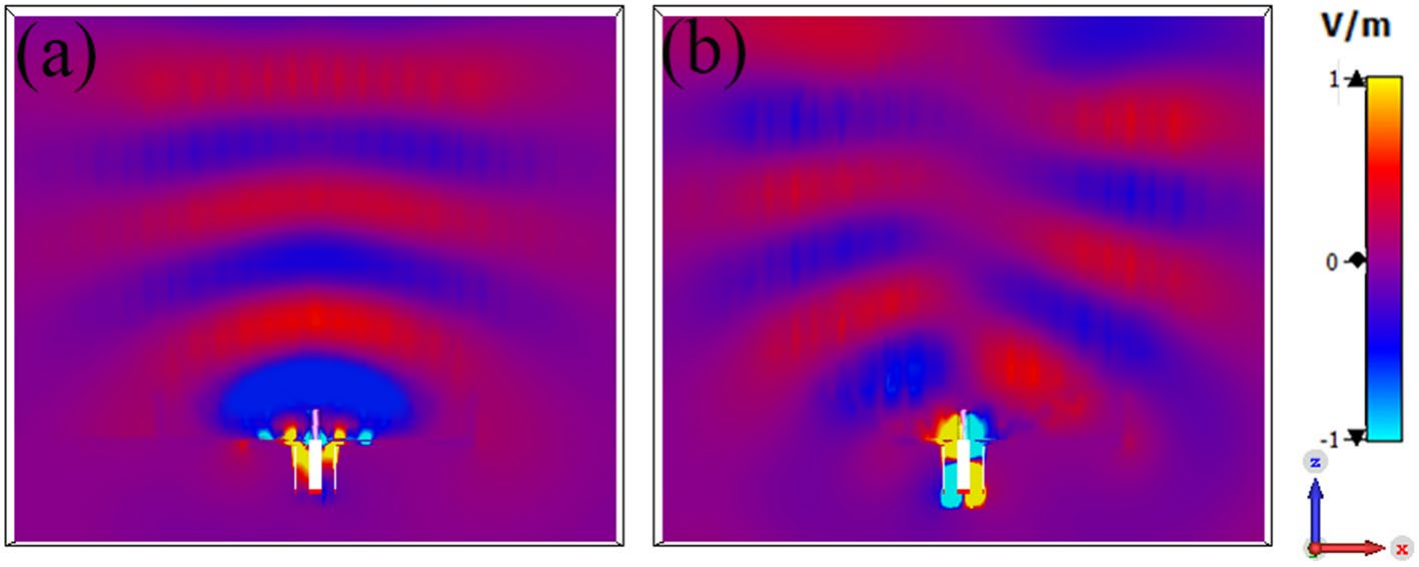
the normalized electric field distribution at 15*GHz*, (**a**) ring patch port, and (**b**) SMA port.

**Figure 17:**
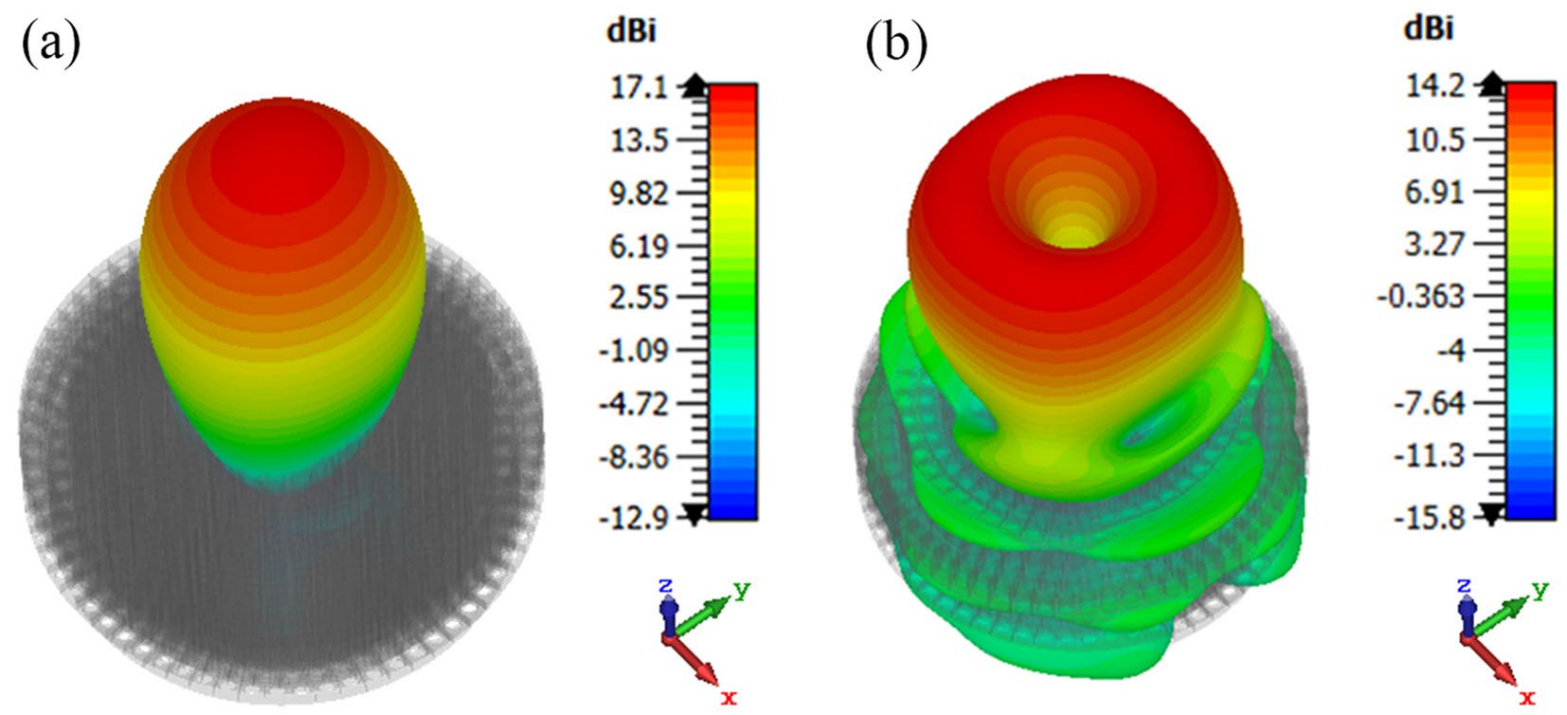
The simulated 3D radiation patterns of the lens antenna at 15*GHz*, (**a**) ring patch port, and (**b**) SMA port.

**Figure 18:**
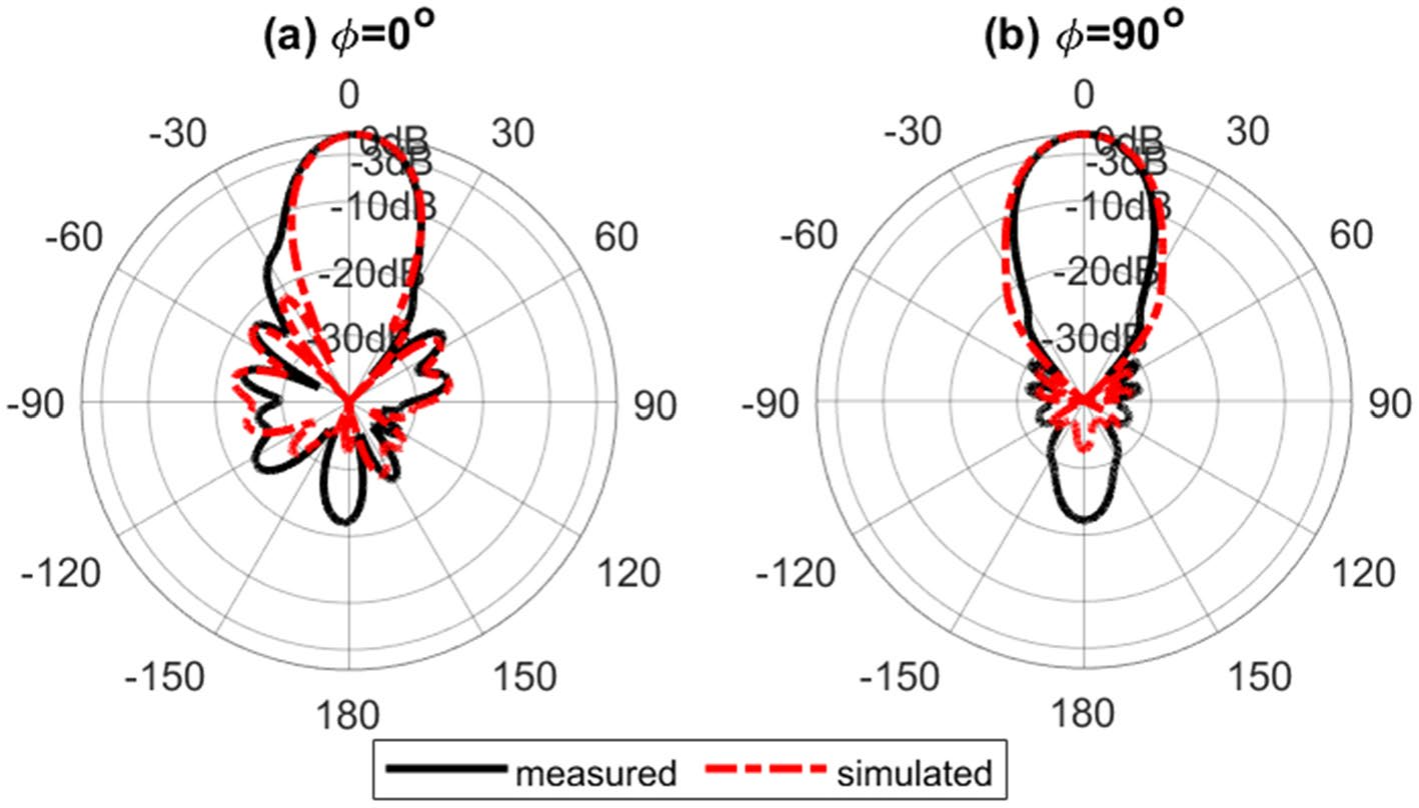
Simulated and measured normalized radiation patterns for the ring patch port on (**a**) $\phi =0^{\circ}$-plane and (**b**) $\phi =90^{\circ}$-plane at 15*GHz*.

**Figure 19:**
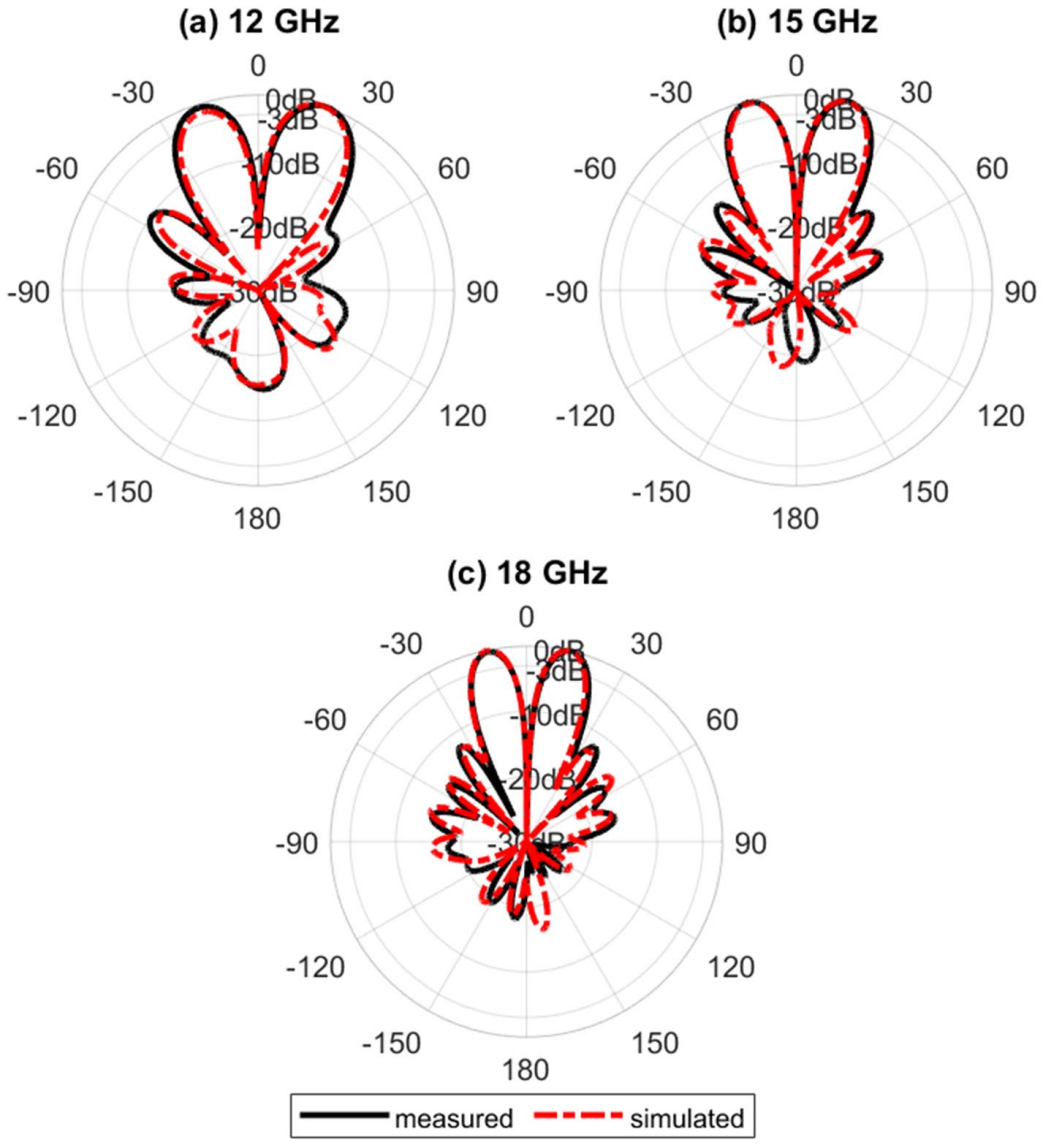
Simulated and measured normalized conical radiation patterns for the SMA port on the $\phi =0^{\circ}$-plane.

**Figure 20:**
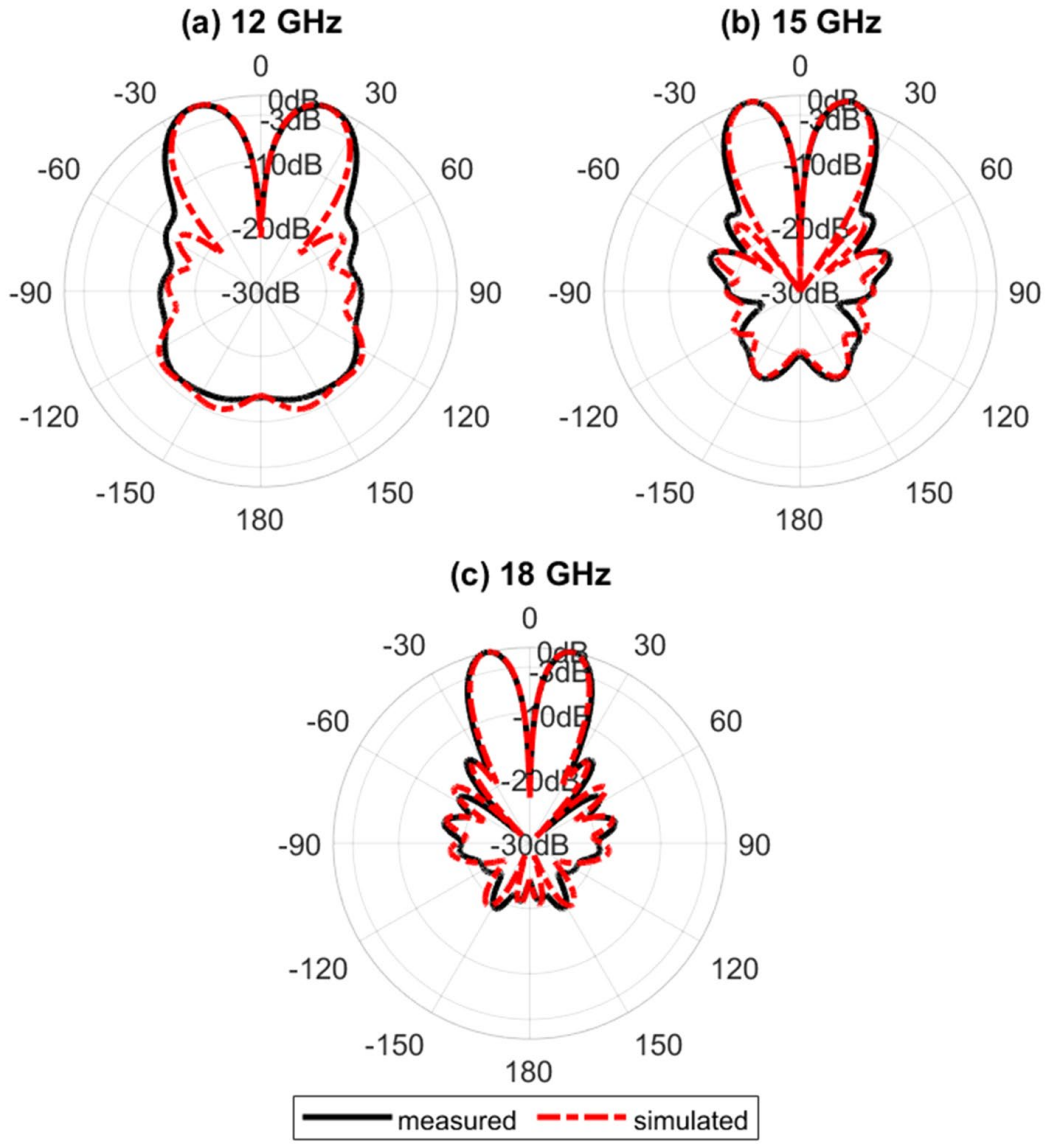
Simulated and measured normalized conical radiation patterns for the SMA port on the $\phi =90^{\circ}$-plane.

**Figure 21:**
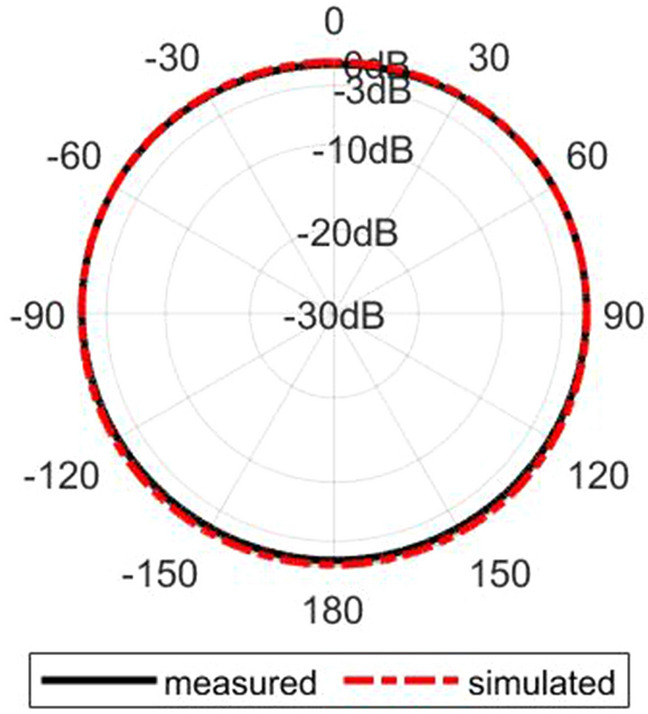
Simulated and measured normalized conical radiation patterns for the SMA port on constant ($\theta =15^{\circ}$)-plane at 15 GHz.

**Figure 22:**
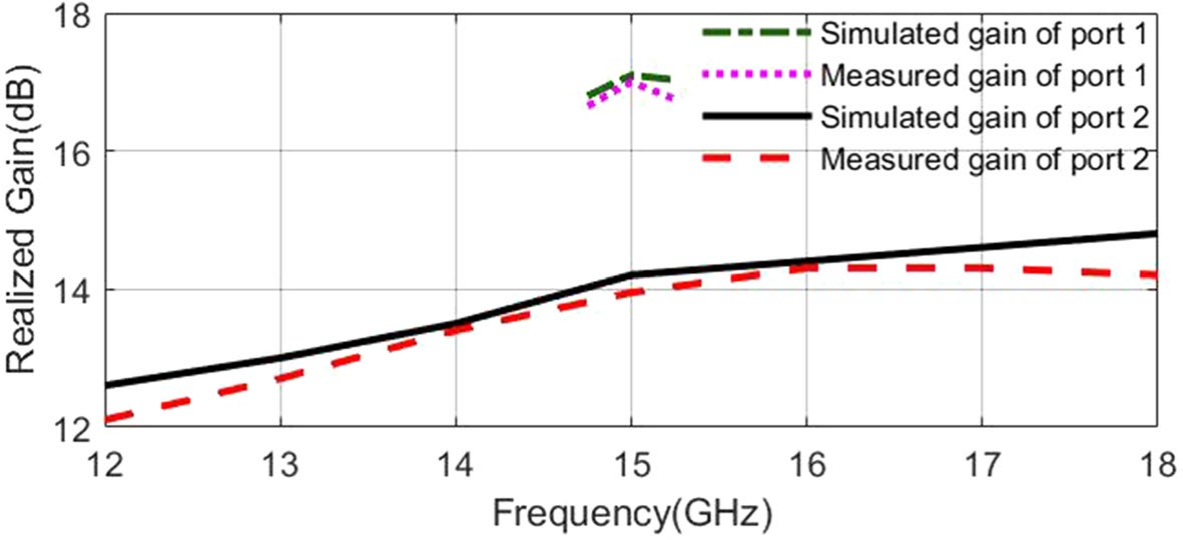
Measured and simulated realized gain for both ports.

Figure 16 shows the simulated normalized electric field distribution at $15$ GHz for the proposed lens. The simulated 3D radiation patterns of the lens antenna are plotted in Fig. 17 at $15$ GHz. The simulated realized gain of broadside and conical radiation patterns are 17.1 and 14.2 dBi, respectively. The co-polarization of the conical pattern is the same as the polarization of the monopole SMA-connector,i.e., linear polarization ($E_\theta$). The polarization of the cross-polarization of the conical pattern is $E_\phi$. The performance characteristics of the lens antenna 2D radiation patterns are shown in Figs. 18, 19, and 20. Figure 18a,b illustrate the simulated and measured normalized radiation patterns for the ring patch port on the $\phi =0^{\circ}$-plane and $\phi =90^{\circ}$-plane at 15 GHz, respectively. The side lobe level (SLL) is lower than $-22$ dB for both planes. The E-plane ($\phi =0^{\circ}$-plane) and H-plane ($\phi =90^{\circ}$-plane) half-power beam widths (HPBW) are 22 degrees and 24 degrees, respectively. Figures  19 and 20 illustrate the simulated and measured normalized conical radiation patterns for the SMA port on $\phi =0^{\circ}$-plane and $\phi =90^{\circ}$-plane at some frequencies, respectively. Also, symmetrical conical radiation patterns with a null at the zenith ($\theta = 0^{\circ}$) are observed for the XZ- and YZ-plane cuts. Figure 21 illustrates the simulated and measured normalized conical radiation patterns for the SMA port on constant ($\theta =15^{\circ}$)-plane at 15 GHz. Conical radiation pattern results indicate that elevation beam coverage is from $7.5^{\circ}$ to $23.5^{\circ}$, and the maximum realized gain is 14.2 dBi at $15.4^{\circ}$ elevation at 15 GHz. Also, measured and simulated realized gain for both ports are depicted in Fig. 22. Good agreement is observed between the simulated and measured radiation patterns. The performance of some reported conical pattern antennas is summarized in Table 3 to compare with that of the proposed work. The advantage of the presented designed structure over other works is its high realized gain and broad bandwidth. Also, a simple SMA connector is used in this paper as a feed of the lens to generate omnidirectional conical patterns instead of complex antenna shapes in other works.

**Table 3:** Performance comparison conical radiation pattern antennas.

Refs.	BW %	$f_0$ (GHz)	Gain dBi	Dimension /$\lambda _0^2$	Height/$\lambda _0$
^1^	$25.6\%$	5.62	6	$1.12 \times 1.12$	0.09
^6^	$13\%$	5.8	4.4	$1.28 \times 1.28$	0.03
^21^	$23.5\%$	2.85	6.9	$1.28 \times 1.28$	0.33
^60^	$16.35\%$	10	5.3	$2 \times 2$	0.052
^61^	$197.5\%$	20	4.8	$7.72 \times 7.72$	3.6
^62^	$4\%$	10	14	$9 \times 1.28$	0.0083
This work	$>50\%$	15	14.2	$2.5 \times 2.5$	1.875

## Conclusion

In this manuscript, an inhomogeneous lens was presented based on the ray-inserting method. A simple circular patch ring and RF connector were used as a feed of the lens to generate broadside and omnidirectional conical patterns, respectively. The presented inhomogeneous lens not only increases the gain of the RF connector monopole antenna and creates a conical-shaped radiation pattern but also can develop a suitable matching in the broad frequency bandwidth. The lens structure is realized and fabricated using the 3D printing method. The broadside realized gain is 17.1  dBi, and its E-plane ($\phi =0^{\circ}$-plane) and H-plane ($\phi =90^{\circ}$-plane) half-power beam widths (HPBW) are 22 degrees and 24 degrees at 15  GHz, respectively. Conical radiation pattern results indicate that elevation beam coverage is from $7.5^{\circ}$ to $23.5^{\circ}$, and the maximum realized gain is 14.2  dBi at $15.4^{\circ}$ elevation at $15\,\,GHz$. The results of simulations and experiments indicate good performances of the designed lens in the wide frequency bandwidth.

## Data Availability

The datasets used and analysed during the current study available from the corresponding author on reasonable request.
